# The role of spinal cord extrasynaptic α_5_GABA_A_ receptors in chronic pain

**DOI:** 10.14814/phy2.14984

**Published:** 2021-08-19

**Authors:** Rodolfo Delgado‐Lezama, Mariana Bravo‐Hernández, Úrzula Franco‐Enzástiga, Yarim E. De la Luz‐Cuellar, Nara S. Alvarado‐Cervantes, Guadalupe Raya‐Tafolla, Luis A. Martínez‐Zaldivar, Alberto Vargas‐Parada, Erick J. Rodríguez‐Palma, Guadalupe C. Vidal‐Cantú, Crystell G. Guzmán‐Priego, Jorge E. Torres‐López, Janet Murbartián, Ricardo Felix, Vinicio Granados‐Soto

**Affiliations:** ^1^ Departamento de Fisiología Biofísica y Neurociencias Cinvestav Mexico City Mexico; ^2^ Neuroregeneration Laboratory Department of Anesthesiology University of California San Diego, La Jolla CA USA; ^3^ Neurobiology of Pain Laboratory Departamento de Farmacobiología Cinvestav Mexico City Mexico; ^4^ Mechanisms of Pain Laboratory División Académica de Ciencias de la Salud Universidad Juárez Autónoma de Tabasco, Villahermosa Tabasco Mexico; ^5^ Hospital Regional de Alta Especialidad “Dr. Juan Graham Casasús”, Villahermosa Tabasco Mexico; ^6^ Departamento de Farmacobiología Cinvestav Mexico City Mexico; ^7^ Departamento de Biología Celular Cinvestav Mexico City Mexico

**Keywords:** extrasynaptic GABA_A_ receptors, GABA_A_ and GABA_B_ receptors, Hoffmann reflex, Pain

## Abstract

Chronic pain is an incapacitating condition that affects a large population worldwide. Until now, there is no drug treatment to relieve it. The impairment of GABAergic inhibition mediated by GABA_A_ receptors (GABA_A_R) is considered a relevant factor in mediating chronic pain. Even though both synaptic and extrasynaptic GABA_A_ inhibition are present in neurons that process nociceptive information, the latter is not considered relevant as a target for the development of pain treatments. In particular, the extrasynaptic α_5_GABA_A_Rs are expressed in laminae I‐II of the spinal cord neurons, sensory neurons, and motoneurons. In this review, we discuss evidence showing that blockade of the extrasynaptic α_5_GABA_A_Rs reduces mechanical allodynia in various models of chronic pain and restores the associated loss of rate‐dependent depression of the Hoffmann reflex. Furthermore, in healthy animals, extrasynaptic α_5_GABA_A_R blockade induces both allodynia and hyperalgesia. These results indicate that this receptor may have an antinociceptive and pronociceptive role in healthy and chronic pain‐affected animals, respectively. We propose a hypothesis to explain the relevant role of the extrasynaptic α_5_GABA_A_Rs in the processing of nociceptive information. The data discussed here strongly suggest that this receptor could be a valid pharmacological target to treat chronic pain states.

## Graphical abstract

The extrasynaptic α5GABAARs expressed in the dorsal horn spinal cord neurons mediate mechanical allodynia in various models of chronic pain. The extrasynaptic α5GABAARs have an antinociceptive and pronociceptive role in healthy and chronic pain‐affected animals, respectively. The extrasynaptic α5GABAARs could be a valid pharmacological target to treat chronic pain syndromes.

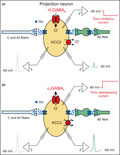

## 1 INTRODUCTION

The circuit processing nociceptive information located mainly in laminae I–III of the spinal cord's dorsal horn contains, among other cells, projection neurons, GABAergic, glycinergic, and glutamatergic interneurons, Aβ, Aδ, and C primary afferent fibers, as well as descending afferent fiber terminals (Todd, 2010). Under normal conditions, projection neurons are activated mainly by nociceptive primary afferent fibers generating a withdrawal reaction from the noxious stimulus. The excitability of projection neurons is regulated by a vast repertoire of channels and receptors, including the GABA_A_ receptors (GABA_A_Rs). Most GABA_A_Rs mediate synaptic communication and are located in the underlying postsynaptic density. These receptors can be activated by GABA release from presynaptic vesicles, increasing the membrane permeability to chloride and bicarbonate ions for brief periods (<100 ms), producing inhibitory (IPSC) or excitatory (EPSC) postsynaptic currents in mature and immature and sensory neurons, respectively. Though a subpopulation of GABA_A_Rs is present in somatic, dendritic, and axonal membranes, they are not in opposition to presynaptic terminals. Extrasynaptic receptors mediate an alternative form of inhibition that modulates the neurons’ excitability by a persistent increase in conductance and tonic shunt (Brickley et al., 1996; Farrant & Nusser, 2005; Kullmann et al., 2005). The transmitter release from nociceptive and non‐nociceptive primary afferents is under presynaptic GABAergic control through the axo‐axonic synapses (Rudomin & Schmidt, 1999). According to the gate theory, this presynaptic inhibition regulates the excitatory input onto projection neurons preventing its activation (Melzack & Wall, 1965).

Based on its pharmacological properties, GABA_A_Rs can be divided into sensitive and nonsensitive to benzodiazepines. This feature is related to the subunit composition of the receptor because the action of these drugs is mediated by a specific binding site located in the α_1,2,3,5_ and γ subunits. GABA_A_Rs containing α_4_ or α_6_ subunits do not bind benzodiazepines and are associated with the δ subunit (Rudolph & Knoflach, 2011; Zeilhofer et al., 2012). The side effects of benzodiazepines, such as sedation and addiction after long‐term use, are considered important issues in developing new GABA_A_Rs subtype‐selective compounds to overcome the limitations of classical benzodiazepines (Zeilhofer et al., 2012).

## 2 GABA_A_ RECEPTORS IN THE SPINAL CORD

Immunohistochemical, in situ hybridization, and functional studies (Alvarez et al., 1996; Bohlhalter et al., 1996; Ma et al., 1993) have demonstrated the expression and localization of α_2,3,5_GABA_A_Rs and δ subunit‐containing GABA_A_Rs in the dorsal horn neurons. In particular, the α_5_GABA_A_R and those containing the δ subunit are expressed extrasynaptically in neurons of laminae I and II mediating a tonic current (Bonin et al., 2011; Perez‐Sanchez et al., 2017; Takahashi et al., 2006; Todd, 2010). Likewise, primary afferent fibers also express synaptic α_2_GABA_A_Rs (Witschi et al., 2011) and extrasynaptic α_5_GABA_A_Rs that mediate a phasic and tonic depolarization, respectively (Bravo‐Hernández et al., 2016; Hernández‐Reyes et al., 2019; Lucas‐Osma et al., 2018). Moreover, α_5_GABA_A_R mRNA and protein are present in sciatic nerve, dorsal root, and dorsal root ganglion (DRG) neurons (Bravo‐Hernández et al., 2016; Loeza‐Alcocer et al., 2013). α_5_GABA_A_Rs are found in primary afferent terminals co‐localizing with peptidergic terminals in lamina IIo, non‐peptidergic terminals in lamina Iii, and with myelinated terminals in lamina III (Paul et al., 2012). Besides, α_5_GABA_A_Rs have been found in glutamatergic and GABAergic neurons of layers II–V of the spinal dorsal horn (Bohlhalter et al., 1996; Ma et al., 1993), motoneurons (Alvarez et al., 1996; Canto‐Bustos et al., 2017), and ventral horn interneurons (Castro et al., 2011). There is evidence that the most profuse subunits along the laminae of the spinal cord are α_3_>α_2_>α_5_>α_4/6_ in combination with γ_2_, β_2/3_, and δ subunits (Alvarez et al., 1996; Bohlhalter et al., 1996; Paul et al., 2012).

## 3 PAMS AND NAMS TO TREAT CHRONIC PAIN

The GABAergic inhibition, mediated by GABA_A_Rs, in the spinal cord is so relevant that its blockade with bicuculline, an antagonist of GABA_A_Rs, produces allodynia and hyperalgesia in healthy rodents (Roberts et al., 1986). Consequently, based on this fact, the development of chronic and neuropathic pain is considered a result of a loss of GABA_A_ inhibition in the spinal cord (Bonin & De Konick, 2013; Munro et al., 2011; Zeilhofer et al., 2012). Many mechanisms have been described to explain the sensitization at the spinal dorsal horn produced by the loss of synaptic GABA_A_ inhibition. One of these studies shows a substantial increase in polysynaptic input onto lamina II neurons in the presence of bicuculline (Baba et al., 2003). Another interesting study was performed by recording second‐order lamina I neurons, which express neurokinin 1 receptors and receive sensory excitatory synaptic inputs exclusively from C and Aδ nociceptors. In the presence of GABA_A_R and GlyR antagonists, polysynaptic inputs appeared in response to Aβ fiber activation (Torsney & MacDermott, 2006). Similar anomalous synaptic inputs were recorded from neurons of the substantia gelatinosa in transverse spinal cord slices from animals with chronic pain (Baba et al., 1999). The presence of this polysynaptic excitatory input has been proposed to underlie allodynia activated in vivo after the intrathecal application of bicuculline or strychnine (Zeilhofer et al., 2015). These studies indicate the relevance of GABA_A_ synaptic inhibition as a pharmacological target for reversing pathological pain states. Taking into consideration, all these evidence by transgenic mice with mutated α subunits, a positive allosteric modulators (PAM) battery of α_2,3,5_GABA_A_R with less sedating benzodiazepines action has been developed to restore the loss of synaptic GABA_A_ inhibition to relieve pain (Bonin & De Koninck, 2013; Knabl et al., 2008; Munro et al., 2011; Zeilhofer et al., 2012). In parallel, the use of point‐mutated mice has shown that the ablation of the α_2_GABA_A_R has a strong antihyperalgesic effect with reduced side effects in different pain models (Ralvenius et al., 2015). In addition, the pharmacological targeting of this receptor could prevent the development of tolerance.

All these studies indicate that the different subtypes of benzodiazepine‐sensitive GABA_A_Rs contribute to spinal antihyperalgesia with the rank order α_2_>α_3_>α_5_>α_1_. The antihyperalgesic action of benzodiazepines has been tested in three pain models, that is, zymosan A, chronic constriction injury of sciatic nerve and formalin test (Zeilhofer et al., 2015). In addition, it has been reported that α_5_GABA_A_Rs activation, via the administration of PAMs, does not induce analgesia in inflammatory and neuropathic pain models (Munro et al., 2011; Zeilhofer et al., 2012).

## 4 PRONOCICEPTIVE ACTION OF α_5_GABA_A_R IN DIABETIC NEUROPATHY

Investigating the mechanisms underlying hyperalgesia developed by the formalin test in diabetic rats, it was found a paradoxical reduction of glutamate, the increase of GABA, and downregulation in the expression of the K^+^‐Cl^−^ co‐transporter 2 (KCC2) in the laminae I‐II of the dorsal horn spinal cord (Jolivalt et al., 2008; Morgado et al., 2008). Unexpectedly bicuculline reduced formalin‐evoked flinching and alleviated tactile allodynia (Jolivalt et al., 2008), suggesting that reduced KCC2 expression in parallel with the increased GABA release contribute to the allodynia and hyperalgesia in diabetes (Jolivalt et al., 2008). The relevant role of GABA_A_Rs in diabetic neuropathy is highlighted by the evidence that in dorsal horn neurons, even when subjected to intense glycinergic inhibition (Yoshimura & Nishi, 1995), the blockade of GABA_A_Rs is enough to reduce allodynia and produce it in healthy animals, respectively. Interestingly, some of the molecular and cellular alterations produced in the spinal cord in diabetic neuropathy are quite similar to that reported in nerve injury models (Coull et al., 2003). In this case, KCC2 is also downregulated by the binding of BDNF to TrK1 receptors producing the depolarization of the E_Cl−_ to a level high enough to generate action potentials when GABA_A_Rs are activated (Coull et al., 2003, 2005). Therefore, KCC2 downregulation in diabetic neuropathy might also produce a switch from GABA_A_ inhibition to the excitation of laminae I‐II neurons involved in the nociceptive processing information as in peripheral nerve injury. Unexpectedly, but in accordance with Jolivalt et al. (2008), our results have shown that the blockade of the α_5_GABA_A_Rs is enough to reverse mechanical allodynia in streptozotocin (STZ)‐induced diabetic rats (Hernández‐Reyes et al., 2019), suggesting that this receptor contribute significantly to pain in diabetic neuropathy.

## 5 THE ANTINOCICEPTIVE AND PRONOCICEPTIVE ROLE OF α_5_GABA_A_R IN CHRONIC INFLAMMATION AND NEUROPATHIC PAIN

It is well known that the tonic current mediated by extrasynaptic GABA_A_Rs has great relevance in physiological and pathological events both at the level of individual neurons and in neural networks (Farrant & Nusser, 2005). Even though the α_5_GABA_A_Rs are expressed in laminae I‐II neurons of the network that processes nociception, their role in chronic and neuropathic pain is unknown. Only one study carried out in *Gabra_5_* ^−/−^ mice shows that these receptors mediate a tonic current in those neurons. This work also concludes that α_5_GABA_A_Rs do not modulate acute nociception but play a pronociceptive role at the late inflammation stages (Perez‐Sanchez et al., 2017). Interestingly, intrathecal administration of L‐655,708 (α_5_GABA_A_R inverse agonist) decreases pain threshold in naïve rats (De la Luz‐Cuellar et al., 2019; Franco‐Enzástiga et al., 2021; Hernández‐Reyes et al., 2019) and mice (Xue et al., 2017). In contrast, L‐655,708 prevents and reverses long‐lasting allodynia induced by formalin, Freund's adjuvant, nerve injury (Bravo‐Hernandez et al., 2016; 2014), and reserpine‐induced pain‐type fibromyalgia (De la Luz‐Cuellar et al., 2019). Indeed, a siRNA against α_5_GABA_A_R reduces reserpine‐induced allodynia in female rats (De la Luz‐Cuellar et al., 2019). Interestingly, as intrathecal L,655‐708 (Hernández‐Reyes et al., 2019), the siRNA against α_5_GABA_A_R induces tactile allodynia in naïve rats confirming that spinal α_5_GABA_A_Rs have an antinociceptive and pronociceptive role in healthy and chronic pain, respectively.

## 6 ROLE OF α_5_GABA_A_R IN PRIMARY AFFERENTS, THE HOFFMANN REFLEX, AND CHRONIC INFLAMMATION

**FIGURE 1:**
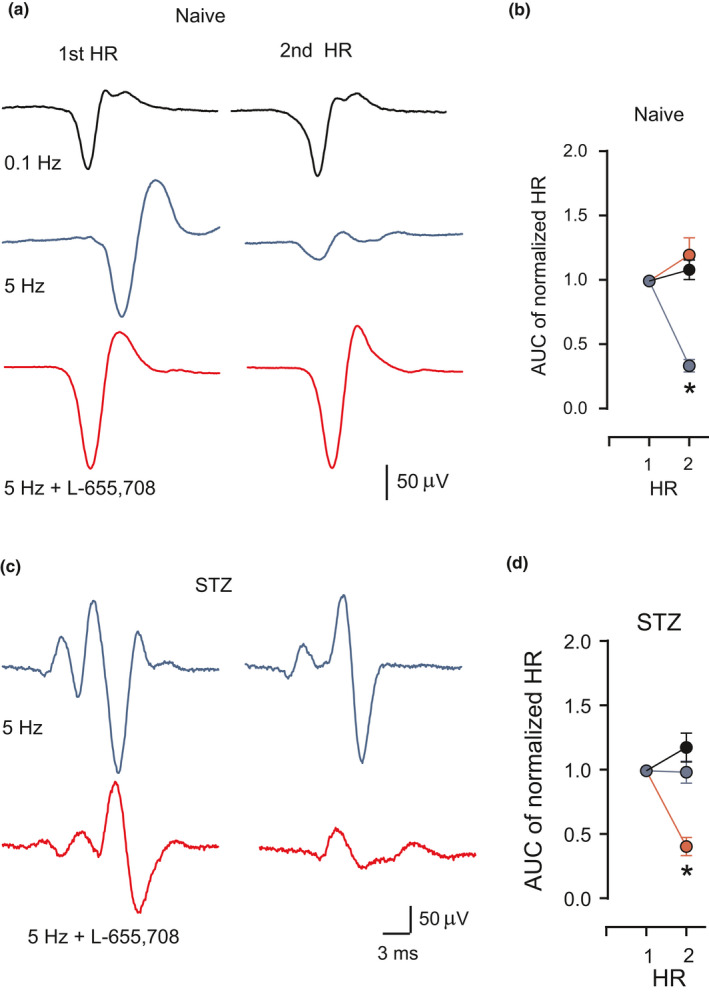
Rate‐dependent depression of the Hoffmann reflex (HR). (a) First and second HR evoked at 0.1 (black trace) and 5 Hz in control (blue trace) condition and after the intrathecal administration of L‐655,708 (red trace) in naïve rats. (b) AUC (area under the curve) of rectified HR2 normalized with respect to the mean area of the first HR at 0.1 Hz (black circles), 5 Hz (blue circles), and 5 Hz in the presence of L‐655,708 (red circles). *p = 0.0001, significantly different with respect to the mean value of HR obtained at 0.1 and 5 Hz (red circle), by 2‐sample Student´s *t*‐test. (c) First and second HR evoked at 5 Hz before (blue trace) and after 2 h (red trace) of intrathecal administration of L‐655,708 in STZ‐diabetic rats. (d) AUC of rectified HR2 normalized with respect to the first HR's mean area at 5 Hz before (blue circles) and after the intrathecal administration of L‐655,708 (red circles). *p = 0.0001, significantly different with respect to the mean values of HR obtained at 5 Hz (blue circle) and 0.1 Hz (black circle) in STZ‐diabetic rats, by two‐sample Student *t*‐test. Data are the mean ± SD from six animals

The α_5_GABA_A_Rs are expressed in primary afferent fibers (Bravo‐Hernández et al., 2016; Hernández‐Reyes et al., 2019; Loeza‐Alcocer et al., 2013; Lucas‐Osma et al., 2018), ventral horn interneurons (Castro et al., 2011), and motoneurons (Canto‐Bustos et al., 2017) where they are activated by the endogenous GABA reducing its excitability tonically. It is well known that the presynaptic inhibition of low threshold primary afferents, associated with its depolarization (PAD), is mediated by the activation of synaptic α_2_GABA_A_Rs (Witschi et al., 2011) and depresses the monosynaptic reflex (MSR) (Rudomin & Schmidt, 1999). Interestingly, L‐655,708 facilitated the MSR without affecting the PAD, suggesting that besides synaptic α_2_GABA_A_Rs also extrasynaptic α_5_GABA_A_Rs are involved in motor control (Canto‐Bustos et al., 2017; Loeza‐Alcocer et al., 2013). The role of α_5_GABA_A_Rs in motor control has been evidenced by the Hoffmann reflex (HR). The property of HR, known as rate‐dependent depression (RDD), has been proposed as a biomarker to detect the presence of neuropathic pain (Marshall et al., 2017). As shown in rats in humans, the RDD is also impaired in diabetic neuropathy (Hernández‐Reyes et al., 2019; Lee‐Kubli & Calcutt, 2014; Marshall et al., 2017). Interestingly, bicuculline restores the loss of RDD, suggesting that the disruption of GABAergic inhibition mediated by GABA_A_Rs is involved in the impairment of this property (Jolivalt et al., 2008; Lee‐Kubli & Calcutt, 2014). We have shown that the intrathecal application of L‐655,708 abolishes RDD in healthy animals and reestablished it in neuropathic diabetic rats in parallel with the reduction of tactile allodynia (Figure 1, Hernández‐Reyes et al., 2019). This result demonstrates that the impairment of RDD is mediated by extrasynaptic α_5_GABA_A_Rs.

There is evidence that formalin‐ or capsaicin‐induced secondary hyperalgesia is associated with the activation of antidromic action potentials in sensory neurons produced by the facilitated PAD (inflammatory conditions). This phenomenon, known as dorsal root reflex (DRR), propagates retrogradely to the peripheral terminal, where it evokes the release of inflammatory neuropeptides (substance P and CGRP) (Willis, 1999), contributing to hyperalgesia (Cervero et al., 2003). In this context, instead of activating GABA_A_Rs, it was proposed to induce analgesia by blocking them. In line with this, the α_5_‐selective negative allosteric modulator (NAM) α5IA‐II was evaluated in the formalin and carrageenan tests. In both cases, it reverses mechanical hypersensitivity and weight‐bearing deficits. In contrast, it did not affect nociception in a neuropathic pain model (Munro et al., 2011). Unexpectedly, in the turtle, we showed that L‐655,708 depresses the DRRs without affecting PAD, suggesting that extrasynaptic α_5_GABA_A_Rs might be tonically depolarizing the primary afferents to reach the threshold to activate the DRRs (Loeza‐Alcocer et al., 2013). In agreement with this result, the peripheral and intrathecal pretreatment or post‐treatment with L‐655,708 prevents and reverses the long‐lasting allodynia and hyperalgesia in the formalin‐induced test and restores the loss RDD of the HR (Bravo‐Hernández et al., 2016).

## 7 HOW SELECTIVE IS L‐655,708?

**FIGURE 2:**
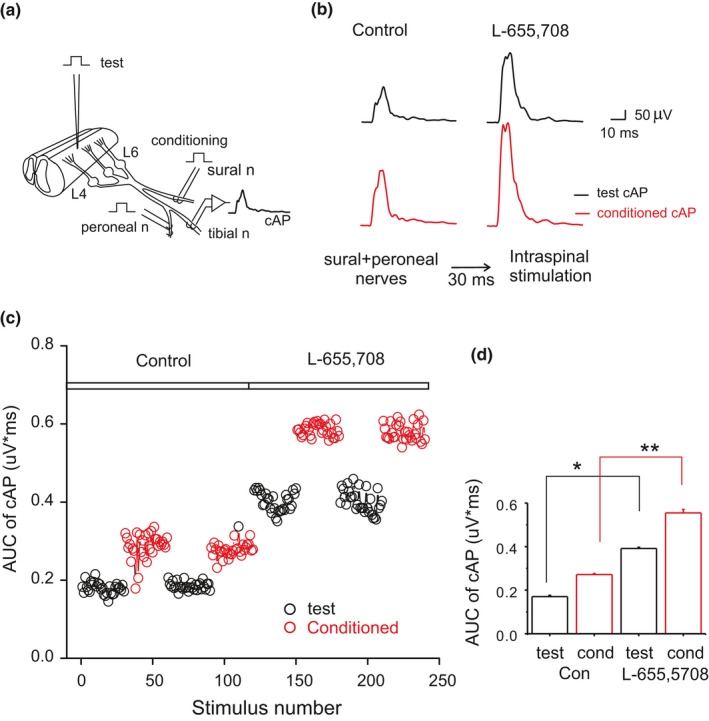
Wall's test to record the phasic and tonic excitability of primary afferent fibers. (a) Scheme showing the spinal cord and the dorsal roots L4‐L6 in continuity with the spinal nerves and the sural, peroneal, and tibial nerves. The spinal cord was electrically stimulated (test) with an electrode place at the lumbar enlargement (L4–L6). The sural and peroneal (conditioning) and tibial nerves are put on a pair of metal electrodes connected the first two to an electric current source and the tibial nerve to the AC amplifier to record the evoked antidromic compound action potential (cAP). (b) Test (black) and conditioned (red) cAP traces recorded before and after intrathecal administration of L‐655,708. (c) AUC of test and conditioned cAP evoked every 5 s recorded in the control condition and after intrathecal administration of L‐655,708. (d) Graph shows the mean area under the curve of test and conditioned cAP recorded in the absence and presence of L‐655,708. *p = 0.0001, by the Student´s *t*‐test. AUC, area under the curve

The pharmacological approach to investigate the function of α_5_GABA_A_Rs has the disadvantage of the differential selectivity of L‐655,708 for the GABA_A_Rs. It displays more selectivity for the α_5_ subunit‐containing receptors than for those containing α_1_, α_2_, α_3_, and α_6_ subunits (Quirk et al., 1996). To circumvent this issue, we have used the excitability Wall‐test in healthy rats. For this, the L1 vertebrae segment was removed to expose the lumbar enlargement, and the antidromic compound action potential (test cAP) recorded in the tibial nerve was evoked by the spinal cord electrical stimulation and was conditioned by the electrical stimulation of the sural and peroneal nerves. The test and the conditioned responses indicate the phasic and the tonic excitability of primary afferent fibers, respectively. Interestingly, the phasic excitability of primary afferent fibers mediated by the activation of synaptic α_2/3_GABA_A_Rs was not affected by L‐655,708, while the tonic excitability mediated by extrasynaptic α_5_GABA_A_Rs was increased (Figure 2; Hernández‐Reyes et al., 2019). Therefore, this result, together with the action of the α_5_GABA_A_R siRNA in healthy and reserpine‐induced fibromyalgia (De la Luz‐Cuellar et al., 2019), confirms the antinociceptive and pronociceptive role of α_5_GABA_A_Rs in healthy and chronic pain conditions, respectively.

## 8 WHY IS THE α_5_GABA_A_R SO RELEVANT IN HEALTH AND PAIN?

**FIGURE 3:**
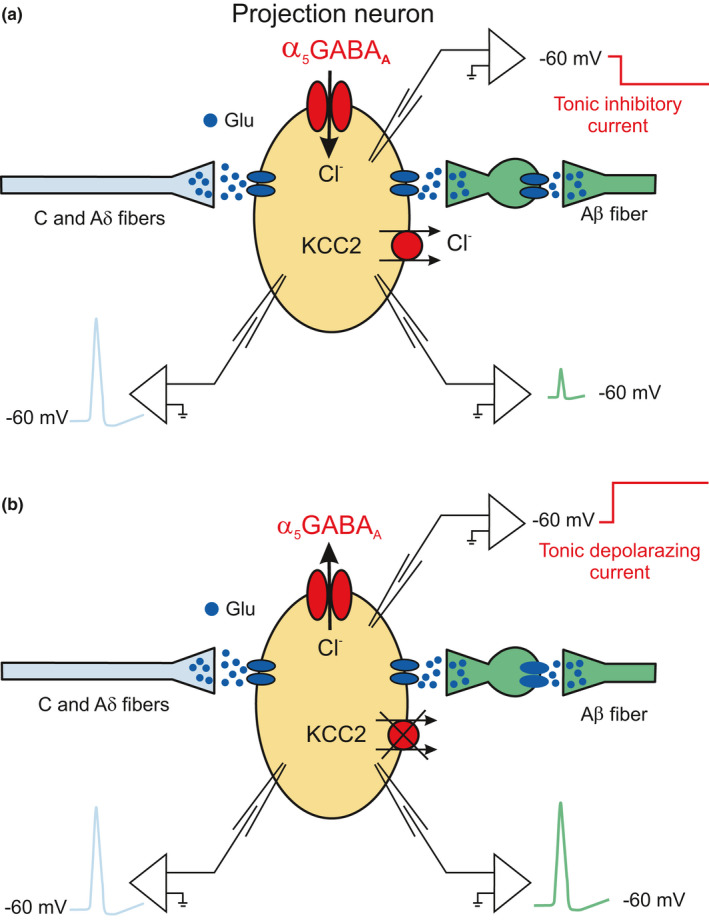
Proposed mechanisms by which the α_5_GABA_A_Rs control the excitability of the projection neurons. In the two schemes C, Aδ, and Aβ primary afferent fibers make mono and polysynaptic connections onto a projection neuron, respectively. (a) In healthy conditions, the α_5_GABA_A_Rs hyperpolarize the projection neurons where KCC2 keeps low [Cl^−^]_i_ preventing Aβ fibers from activating action potentials in the projection neurons. (b) In the chronic pain condition, down‐regulation of KCC2 depolarizes the E_Cl−_ switching α_5_GABA_A_Rs from inhibition into tonic excitation, allowing Aβ fibers to generate action potentials in the projection neurons

α_5_GABA_A_Rs are expressed in laminae I and II neurons, where they mediate a tonic inhibitory current in healthy animals (Perez‐Sanchez et al., 2017). KCC2 is downregulated in the dorsal horn of rodents with nerve injury neuropathy (Coull et al., 2003) and diabetic neuropathy (Jolivalt et al., 2008). Therefore, the action of GABA_A_R might be switching from inhibition into excitation, which has been shown in nerve injury (Coull et al., 2003). Given that the action of extrasynaptic GABA_A_Rs is about six times more intense than the synaptic receptors (Ataka & Gu, 2006), we have hypothesized that the extrasynaptic spinal α_5_GABA_A_Rs might be tonically hyperpolarizing the projection neurons in the health condition, while in chronic pain they might be causing a tonic depolarization of the cell membrane. In the first case, they prevent the excitatory synaptic inputs of the low threshold afferent fibers from activating the projection neurons of laminae I‐II (Baba et al., 2003; Torsney & MacDermott, 2006), and in the second condition, the gate opens, allowing the fibers mentioned above to activate the neurons (Baba et al., 1999; Figure 3).

Although we have shown that α_5_GABA_A_Rs have a pronociceptive role in several models of chronic pain, there remains an unanswered question: after blocking these receptors, what inhibits the projection neurons, preventing them from being stimulated by the activation of the low‐threshold afferent fibers? One possibility is the participation of the GABA_B_ receptors (GABA_B_Rs). The expression of GABA_B_Rs in dorsal horn neurons and primary afferent fibers in the spinal circuitry processing nociceptive information is well documented (Malcangio 2018; Schuler et al., 2001; Towers et al., 2000). Indeed, GABA_B_Rs are tonically active inhibiting transmitter release (Peshori et al., 1988). At postsynaptic neurons, they control neuronal excitability. Moreover, activation of these receptors with baclofen inhibits the plateau properties, wind‐up, and post‐discharge induced by dorsal root stimulation in dorsal horn neurons even in the presence of tetrodotoxin (Russo et al., 1998) or by noxious mechanical stimulation in vivo (Reali et al., 2011). Lee‐Kubli et al. (2021) confirmed that the GABA_B_Rs in conjunction with the GABA_A_Rs contribute to the inhibitory circuitry involved in the modulation of RDD of the HR and tactile allodynia in diabetic rats (Lee‐Kubli et al., 2021).

Interestingly, phaclofen, an antagonist of the GABA_B_Rs, but not bicuculline, impaired the RDD in 4‐week diabetic rats, suggesting that in these animals the normal RDD is mediated by functional GABA_B_Rs. It is well known that in 8‐week diabetic rats, bicuculline and L‐655708 restored the impaired RDD and reversed the mechanical allodynia (Hernández‐Reyes et al., 2019; Jolivalt et al., 2008). However, unexpectedly, the administration of phaclofen in these rats reverted the restoration of RDD by bicuculline. In addition, the intrathecal application of baclofen alleviated mechanical allodynia in diabetic rats. These results suggested to the authors that GABA_B_Rs, together with GABA_A_Rs, are mediating the GABAergic inhibition in the dorsal horn of the spinal cord where nociceptive information is processed. Consequently, if GABA_B_Rs are closing the gate to the excitatory input of low‐threshold afferent fibers on projection neurons, it would be interesting to know whether intrathecal administration of a GABA_B_ receptor antagonist restores mechanical allodynia in diabetic rats after having removed it by blocking α_5_GABA_A_Rs receptors.

## 9 EXTRASYNAPTIC α_5_GABA_A_ RECEPTORS REGULATION

Previous pharmacological studies have reported sex differences in α_5_GABA_A_Rs function. PAMs of α_5_GABA_A_Rs reduce stress‐induced behaviors in females but not in male rodents (Piantadosi et al., 2016). More recently, our group reported a sex‐dependent effect of α_5_GABA_A_Rs activation in chronic pain (De la Luz‐Cuellar et al., 2019; Franco‐Enzástiga et al., 2021). We found that α_5_GABA_A_Rs mRNA and protein changes in DRG and spinal cord are modulated in a sex‐dependent way. Nerve injury increases the expression of α_5_GABA_A_R in female DRG and spinal cord in female rodents, but not in males (Franco‐Enzástiga et al., 2021). These changes are activated by DNA methylation in the CpG island of *Gabra5* in males but not females. In contrast, the expression and function of spinal α_5_GABA_A_Rs in females is associated with the presence of 17β‐estradiol (Franco‐Enzástiga et al., 2021).

## 10 CONCLUSIONS

Multiple and irreversible plastic changes involving non‐neuronal and neuronal cells in the spinal cord circuits processing nociceptive information convey by sensory neurons underlie chronic and neuropathic pain. Between the complexity of adaptations in the function of the nervous system, the loss of inhibition mediated by synaptic GABA_A_Rs has been identified as a target for developing drugs to relieve pain. Reestablishing GABAergic inhibition without knowing where and how the inhibition is lost represents a very complex issue. The reversion of allodynia by blocking the α_5_GABA_A_Rs in pain induced by diabetes, nerve injury, fibromyalgia, and chronic inflammation indicates that the spinal inhibitory dysfunction might be related to a downregulation of KCC2 transporter affecting the homeostasis of Cl^−^ in projection neurons, where the tonic current through this receptor might be switched from inhibition to excitation. Therefore, blocking α_5_GABA_A_Rs may be a feasible strategy to treat chronic pain.

## CONFLICTS OF INTEREST

The authors declare no conflict of interest.

## AUTHOR CONTRIBUTIONS

All authors read, improved, and approved the manuscript. V.G.‐S., and R.F., and R.D.‐L., wrote the manuscript.
